# Modular Morphing Lattices for Large-Scale Underwater Continuum Robotic Structures

**DOI:** 10.1089/soro.2022.0117

**Published:** 2023-08-09

**Authors:** Alfonso Parra Rubio, Dixia Fan, Benjamin Jenett, José del Águila Ferrandis, Filippos Tourlomousis, Amira Abdel-Rahman, David Preiss, Jiri Zemánek, Michael Triantafyllou, Neil Gershenfeld

**Affiliations:** ^1^ Center for Bits and Atoms of USA, Massachusetts Institute of Technology, Cambridge, Massachusetts, USA.; ^2^ Intelligent and Informational Fluid Mechanics Laboratory, Westlake University, Hangzhou, China.; ^3^ Discrete Lattice Industries, LLC, Laguna Beach, California, USA.; ^4^ Sea Grant and Mechanical Engineering Department, Massachusetts Institute of Technology, Cambridge, Massachusetts, USA.; ^5^ Biomolecular Physics Laboratory, Demokritos Research Center, Athens, Greece.; ^6^ Czech Technical University in Prague, Prague, Czech Republic.

**Keywords:** underwater, morphing structures, modular metamaterials, continuum robots

## Abstract

In this study, we present a method to construct meter-scale deformable structures for underwater robotic applications by discretely assembling mechanical metamaterials. We address the challenge of scaling up nature-like deformable structures while remaining structurally efficient by combining rigid and compliant facets to form custom unit cells that assemble into lattices. The unit cells generate controlled local anisotropies that architect the global deformation of the robotic structure. The resulting flexibility allows better unsteady flow control that enables highly efficient propulsion and optimized force profile manipulations. We demonstrate the utility of this approach in two models. The first is a morphing beam snake-like robot that can generate thrust at specific anguilliform swimming parameters. The second is a morphing surface hydrofoil that, when compared with a rigid wing at the same angles of attack (AoAs), can increase the lift coefficient up to 0.6. In addition, in lower AoAs, the L∕D ratio improves by 5 times, whereas in higher angles it improves by 1.25 times. The resulting hydrodynamic performance demonstrates the potential to achieve accessible, scalable, and simple to design and assemble morphing structures for more efficient and effective future ocean exploration and exploitation.

## Introduction

For the past century, considerable energy has been expended in exploring and exploiting the ocean environment, manifested by the progress in the booming marine industries for transportation, renewable energy harvesting, environmental monitoring, and aquaculture farming.^1–4^ However, even today, >80% of the ocean territory is still unmapped, unobserved, and unexplored,^5^ mainly due to the substantial technical challenges that ocean vehicles and structures face when operating in physically, chemically, and biologically harsh aquatic environment. These challenges include intense pressure in the deep water, large unsteady forces within the water medium due to waves, currents, turbulence, and corrosion.

In addition, the ocean itself faces unprecedented challenges and the health of the ocean is under unprecedented threats from rising sea temperature,^6^ ocean acidification,^7^ and plastic pollution.^8^ Therefore, it is critical for the scientific community to develop new safe,^9^ robust,^10^ economically sensible,^11^ and environmentally friendly solutions^12,13^ to explore, monitor,^14^ and exploit the ocean more efficiently and effectively.

One option is to take inspiration from nature.^15^ Compared with traditional aquatic transportation methods, nature provides alternative solutions, proving to be more agile and effective in overcoming aquatic environmental constraints. Studies with live fish^16–18^ and biorobotic devices^19–21^ have demonstrated that there are some areas with a large performance gap^22,23^ between the man-made machines and marine animals. For example, the vortical wake behind a rigid hull submarine imparts energy losses due to flow separation, which results in a significant loss of propulsive efficiency.^24^

In contrast, fish employ flexible actuation that can reduce separation and recover losses incurred by their body, and even harness energy from oncoming unsteady flow.^25^ Compared with the traditional rigid-body vehicles powered by propellers and waterjets, a major difference and defining property in natural underwater propulsion is flexibility. Although the flexibility varies widely across fish and marine mammals,^26^ different studies have commonly shown that the passive and active control of the flexible body and appendages can result in enhanced propulsive efficiency and improved force profile manipulation.^27,28^

Having identified the benefit of a flexible body in improving hydrodynamic performance, especially in transient conditions and within a turbulent flow, several researchers have integrated such a concept in designing new swimming prototypes with classic mechanical approaches. One successful example is the MIT RoboTuna^29^ that demonstrated the power laws that govern efficient unsteady propulsion and demonstrated drag reduction due to active flow control. However, unlike nature that can obtain cohesive single bodies with complex integrated subsystems,^30^ classic mechanical approaches generate independent systems, and it requires time and resources to integrate and operate them. Taking RoboTuna as an example, it was composed of >3000 unique parts delicately assembled with high cost, time, design, and labor penalties.

Recently, soft robotics emerged as an alternative to classic mechanical approaches. The flexible-compliant materials^31^ have been shown to obtain similar mechanical properties as those in nature, which makes mimicking nature-like muscular hydrostats-like behaviors possible. Multiple examples have demonstrated success in different fields, including an inflatable large-scale soft robot that can move between obstacles by growing,^32^ a modular robotic system that is capable of reconfiguring its shape and task,^33^ and an almost indestructible soft walking robot that can resist fire and ice.^34^

Similarly, various examples have showcased better environmental adaptability for soft robotics in the aquatic environment. Researchers have proposed less costly techniques to build aquatic robotic systems, such as casting silicone with intricate geometries. These new fabrication methodologies have been applied to build novel soft manipulators for underwater sampling,^35^ robotic fish showing better swimming efficiency,^36^ and capable of agile maneuvering.^37^ Furthermore, by adding dielectric elastomers as “muscles,” researchers have demonstrated a self-powered underwater robot that can reach a swimming speed of 0.7 body length per second^38^ and withstand a significant hydrostatic pressure in the Mariana Trench.^39^

However, all the aforementioned soft robot applications in the aquatic environment share a similar size at a 0.1 m scale. The construction of such robots would face significant design, fabrication, and control challenges when scaling up to the meter scale. One reason behind the difficulty is the size of the tooling required for fabrication. Tooling scales with the size of the part, as does cost, which prevents rapid iteration or adaptation. In addition, although several researchers have used additive manufacturing, such as 3D printing, to build soft robot prototypes with complex geometry,^40^ achieving larger-scale structures is still a major challenge.

Moreover, 3D printing may introduce additional technological issues, such as undesired anisotropies, greater potential for defects over larger areas, and unfavorable cost scaling of machine hardware.^41^ Therefore, although current soft robot applications have shown promise in building small-scale aquatic instruments, it is challenging to implement similar technologies to withstand large hydro-loads for large-scale soft aquatic robots and appendages with active morphing surfaces offshore and shipping applications.

It is to be noted that treating soft versus hard structures as two extreme opposites is a false dichotomy, as several flexible robots have shown to take advantage of both soft and hard components^42,43^ in their operations. Therefore, in this study, we intend to address the need for producing scalable, low-cost, and high-performance structural systems for flexible aquatic robotic applications using low-density–high-specific stiffness $\left(E^{*}\right)$ cellular structures that assemble as mechanical metamaterials.^44^ Specifically, based on the reversible assembly of discrete modular units to build larger functional structures, this approach has resulted in ultralight lattice materials with record-setting mechanical properties,^45^ large-scale reconfigurability,^46^ and low cost with high repeatability through its use of best-practice manufacturing techniques.^47^

This article presents a novel method to construct larger flexible cellular robots for marine applications. By combining two simple regular part types (rigid and compliant), we show a new design method for high-performance anisotropic lattice structures. These structures are composed of heterogeneous unit cells with custom-designed mechanical anisotropies that can mechanically morph while behaving structurally efficient to external hydrodynamic forces. To demonstrate the validity of our proposed technology, we describe the design, control, fabrication, and tow tank testing of two different robots, a 1.5 m underwater snake and a 0.675 m span morphing wing that can continuously morph to improve their hydrodynamic behavior.

## Materials and Methods

### Discretely assembled mechanical metamaterials

We build upon our past research on discrete assembly of mechanically tunable lattices^48^ and discretely assembled mechanical metamaterials^44^ to generate robotic structures with custom degrees of freedom (DOFs).

**FIG. 1.**
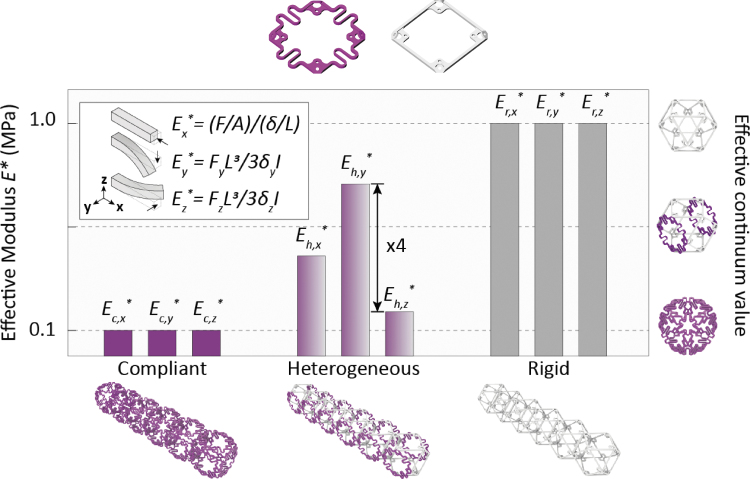
*Upper left*, voxel stiff facet. *Upper right* voxel compliant facet. Comparison of effective axial (x) and bending (y, z) modulus for compliant, heterogeneous, and rigid beams, measured experimentally on 6×1 beams.

We refer to the unit cell as a *voxel*, which is a cuboctahedron decomposed in six faces, as shown in Figure 1. We first build a custom voxel with anisotropic mechanical properties, using compliant and a stiff face. Later, we assemble voxels generating both beam-like and surface-like lattice structures and last, we actuate them internally, generating robots with continuum deformations.

### Selected voxel morphological and mechanical properties

We manufacture the rigid (gray) and compliant (purple) parts by injection molding of Zytel 70G33L (PA6 with 33% of short glass fiber) for the stiff face and Nylon66 for the compliant. When assembled, the lattice has the same pitch of 75 mm, relative density, and cross-section side length of 2.1 by 2.1 mm. The rigid lattice has an effective elastic modulus $E_{R}^{*}$ of 8 MPa, an effective Poisson's ratio of 0.15, and a density of 30 $kg∕m^{3}$. The compliant lattice has an effective elastic modulus $E_{C}^{*}$ of 0.1 MPa, an effective Poisson's ratio of 0.1, and a density of 30 $kg∕m^{3}$.

We performed uniaxial compression tests to measure the axial stiffness of the rigid, compliant, and hybrid voxels. Figure 1 shows that the axial stiffness of the compliant and heterogeneous beams are 11% and 44% of the rigid beam, which demonstrates the effect of heterogeneity producing a value between the two constituent homogeneous values. We then performed linear elastic tip deflection tests of the cantilever beams to calculate their bending stiffness. Figure 1 shows that the bending stiffness of the compliant beam is 13% of the rigid beam, which indicates a good correlation between effective stiffness $E_{R}^{*}$ governing the relationship. We test the heterogeneous beam in both compliant and rigid directions and find that the bending stiffness is 19% and 74%, respectively, which shows that the bending stiffness is strongly anisotropic due to the heterogeneity.

We now take advantage of the mentioned anisotropy to transform the beam into a tendon-driven bending actuator. In the absence of external loads, the neutral surface of tendon-driven beam deflects with a constant curvature.^49–53^ To select a motor that can fit inside a voxel and provide enough torque, we developed an analytical model using a nonlinear beam model solver. We used this model to calculate the centroid radii for arbitrary beam thickness, voxel size, and tendon strain values and then determine the axial tension on the tendon.^53^

(1) $$\kappa =\frac{1}{R_{c}}=\frac{d}{EI^{*}}T,$$

where κ is the curvature (the inverse of the radius $R_{c}$), d is the radial distance from the tendon to the centroid, $EI^{*}$ is the specific bending stiffness, and T is the axial tension value of the tendon. Results obtained from the validation experiments in air are shown in the Supplementary Data.

Given these methods, we now provide two underwater robotic examples capable of both static and dynamic continuum deformations:

(1) A one-dimensional “Hydrosnake” (morphing beam), a slender bioinspired swimming device serves as a platform to show an economical large-scale continuum robot with minimal DOF and unique parts.
(2) A two-dimensional camber “Morphing Foil” (morphing surface), a discrete solution to generate camber active morphing lifting surfaces to maximize the lift-to-drag ratio ($C_{L}∕C_{D}$).

### Hydrosnake

#### Design and fabrication

**FIG. 2.**
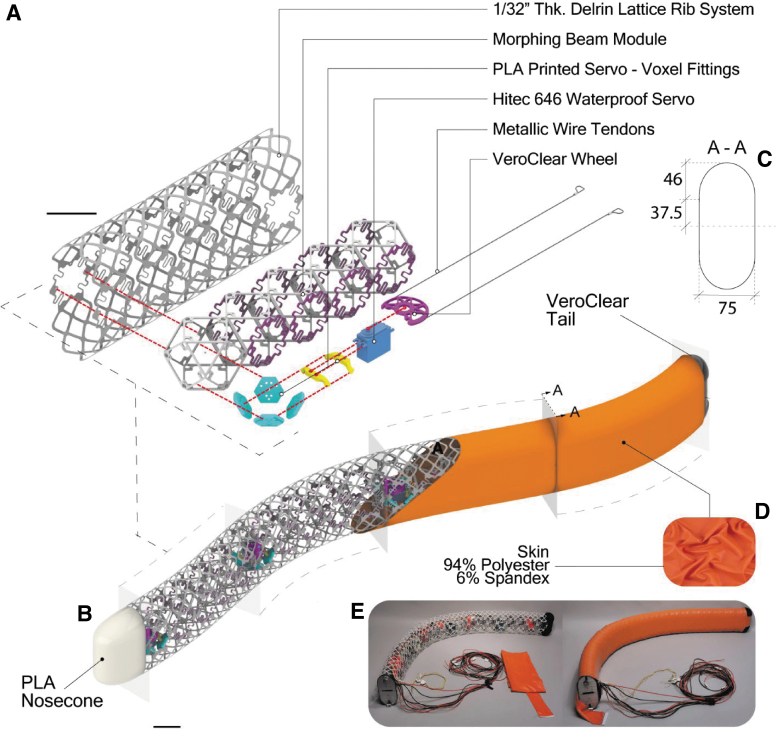
The architecture of the hydrosnake robot. **(A)** Exploded-view of a section. The robot is composed of four of these elements in series. **(B)** Isometric view of the robot highlighting main parts. **(C)** Cross-section drawing. **(D)** Texture of the skin fabric. **(E)** Prototyped robot with and without skin. Scales: **(A)** 75 mm **(B)** 75 mm.

The hydrosnake is a 1.5 m snake-like swimming robot that consists four beam modules as shown in Figure 2. Each module has one rigid and four heterogeneous voxels. A servo is mounted inside the rigid voxel, with two tendons extending to the end of the heterogeneous section. The servo is controlled to rotate the servo horn that translates motor torque τ into a tensile force $F_{t}=\tau ∕r$, where r= 30 mm is the radius of the servo horn.

**FIG. 3.**
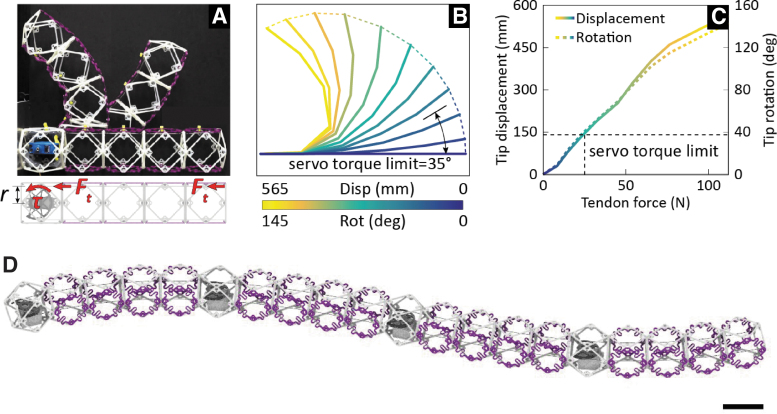
Active morphing heterogeneous beam. **(A)** Morphing beam unit, with undeformed and deformed shape, **(B)** *centerline* positions from tendon-actuated force-displacement controlled testing, and **(C)** force-displacement controlled testing results, with indication of waterproof servo limits. **(D)** 1.5 m discrete continuum soft robot consisting of four morphing beam units connected in series, scale bar = 75 mm.

In Figure 3, we show results from testing actuation force versus tip displacement and rotation. Although it is possible to achieve nearly 180° of tip rotation, this requires roughly 125 N of force, which exceeds the torque capacity of the selected servomotor. Currently, our actuated deformation limit is ∼35° of rotation.

The skin system is composed of an inner lattice rib system and an outer elastic fabric. The ribs, shown in Figure 2A, describe the outer mold line whose cross-section is fabricated as two arches tangent to the parallel compliant faces of the voxel beam. The rib modules can slide past each other to avoid interference during morphing. In addition, we deploy a tailored sewn fabric (94/% polyester and 6% spandex) with a smooth surface finishing. We slide the skin onto the robot as a sock, fix it internally with Velcro on both ends of the body and close the front and rear strips with lateral zippers. The skin is pretensioned in every axis, which prevents the skin from wrinkling or pinching in one tension-compression cycle when the hydrosnake is actuated, shown in Figure 2D. Additional details of the skin and rib design are presented in Supplementary Data.

#### Control of kinematics

In this study, our robot employs a varying amplitude backward-traveling wave to imitate anguilliform swimmer^54^ as follows:

(2) $$y\left(x,t\right)=\frac{A_{t}}{1-e^{\alpha L}}\left(1-e^{-\alpha x}\right)sin\left(\frac{2\pi }{\lambda }x-2\pi ft\right),$$

where y(x,t) is the y coordinate position, L corresponds to the robot length, the tail amplitude $A_{t}$ is chosen from 0.15 to 0.35 L, the vibration frequency f is selected from 0.15 to 0.25 Hz, and α=1.5 controls the conical shape in which the amplitude grows from the leading edge to the trailing edge of the robot.

**FIG. 4.**
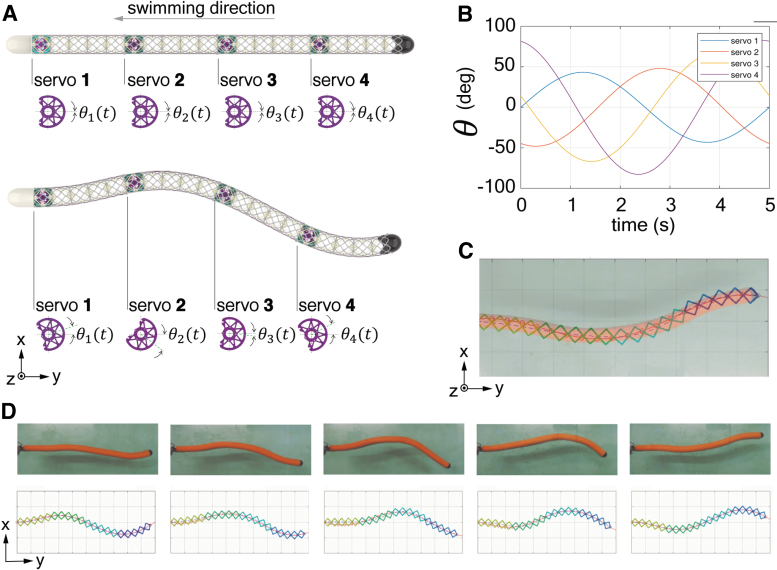
Section-composition of the swimming robot. **(A)** Continuum curvature sections in series replicates target splines. **(B)** Servo actuation phases over time. **(C)** Matching simulation with actuation in a quasi-static state. **(D)** Dynamic matching.

When the internal tendon actuates the robots, their shape/curvature is bounded by the string's maximal contraction divided by the segment length.^55^ The hydrosnake consists of four modules of constant curvature connected in series. We formulate an optimization problem for the robot motion that finds control values for all servos. The control minimizes the distance between the desired shape and the snake's actual shape. Figure 4 shows the comparisons between the commanded body kinematics and the experimental observations.

### Morphing foil

#### Design and fabrication

We build a camber morphing wing using the same voxel geometry as the hydrosnake. The dimensions of the wing are 675 mm span and 508 mm chord, which correspond to a rectangular pattern arrangement of four by nine voxels. We built a custom airfoil by modifying an Eppler 838^56^ to increase its thickness to a 21% and to delay the boundary layer separation under water.

**FIG. 5.**
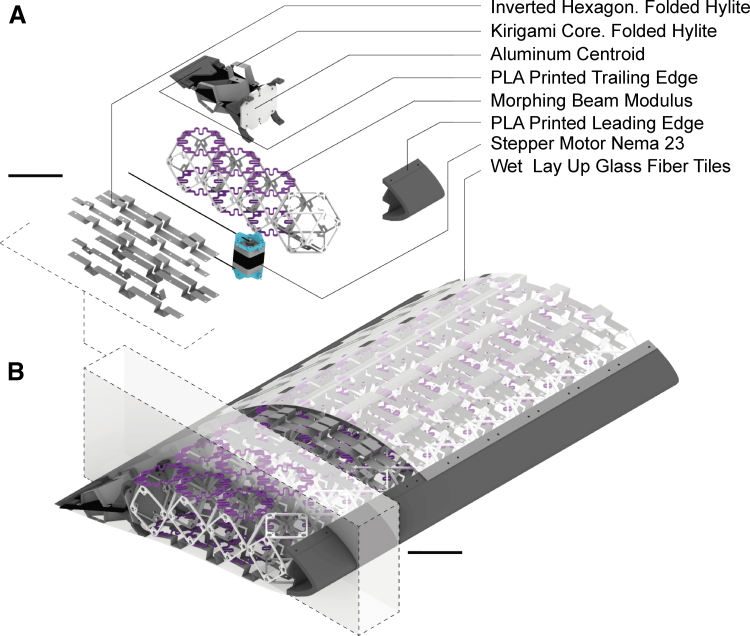
Digital morphing wing system architecture. **(A)** Exploded isometric view of a wing section. Scale 75 mm. **(B)** Isometric view of the full assembly. Scale 75 mm.

The wing structure is composed of four main sections: leading edge, morphing region, trailing edge, and skin. As Figure 5 shows, the leading edge consists of nine polyactic acid 3D printed pieces. Altogether, the pieces encapsulate the aluminum beam that connect the wing with the load cell and the tow tank carriage. It is assembled with the morphing section, composed of one row of rigid voxels and three heterogeneous. The wing is actuated using three NEMA23 dual-shaft stepper motor evenly distributed along the span.

The stepper motors pull stainless steel threaded wires attached to the trailing edge's aluminum rib. The trailing edge is a rigid structure composed by a metallic centroid, and two symmetric kirigami folded structures that provide shape and attach the tiled glass fiber skin. For this morphing robot, precisely controlling the deformed state of the skin was crucial. That would permit us to generate the desired airfoil shapes. That is the reason why we designed curved prestressed composite tiles that would slide respect of each other while keeping a continuous outer surface.

The description of how we obtain analytical deformed shape of the wing can be found in the Supplementary Data.

**FIG. 6.**
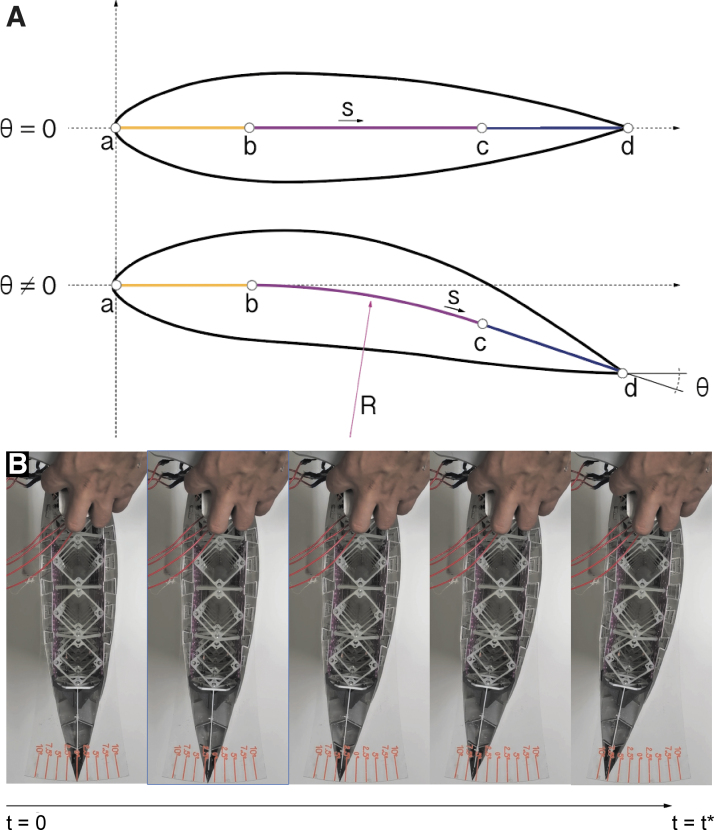
**(A)** Centroid curve parametrization for θ=0 and θ≠0. **(B)** Wing achieving θ=7.5deg.

Compared with the need of the hydrosnake for the dynamical actuation, the morphing foil requires an accurate execution of shape change and is capable of withholding large external force to keep its shape. Therefore, we selected dual-shaft Nema23 stepper motors as the actuator that is able to pull bidirectionally a heterogeneous beam with fine resolution and hold torque with sustainable power consumption increase. It is noted that the stepper motors were assembled inside the first layer of rigid voxels, pulling tendons to an aluminum frame that encapsulates the whole heterogeneous construction. A system to individually prestress the tendons was needed to guarantee that backlash will not affect the performance. The foil is able to perform up to 12.5° rotation continuously, shown in Figure 6A.

## Results and Discussion

### Towing tank test

**FIG. 7.**
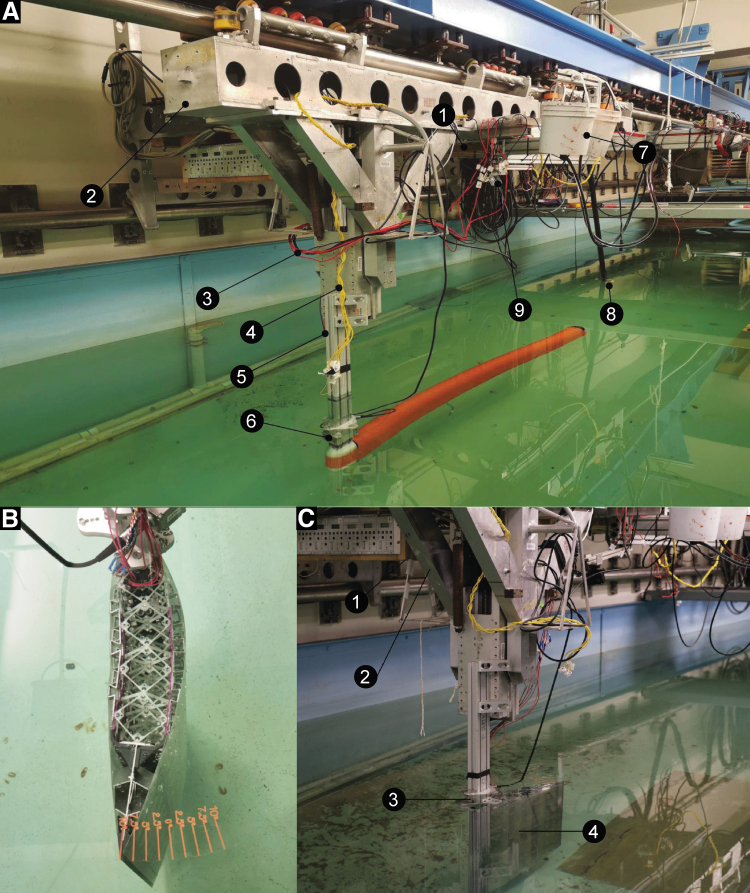
**(A)** Tow tank experiment setup for hydrosnake. (1) Control center zone. Dell computer, Arduino mega microcontroller, ATI gamma load cell amplifier, NI USB-6218 DAQ board and power supplies; (2) tow tank carriage; (3) power lines from fuses to servos; (4) signal lines from microcontroller to servos; (5) snake–carrier fitting; (6) load cell; (7) ink system; (8) water lights; (9) camera. **(B)** Wing in the water at $\theta =10^{o}$ **(C)** tow tank experiment for the morphing wing. (1) Control center (same configuration as image **(A)**). (2) Tow tank carriage. (3) Load cell. (4) Morphing wing. DAQ, data acquisition.

We tested the hydrosnake and wing model at the MIT Towing Tank facility,^57^ which can achieve a steady linear motion from U= 0.05 to 1.5 m/s. Shown in Figure 7a and b, an ATI underwater gamma load cell is mounted between an 8020 aluminum strut and the towing object. The power, control, and data acquisition (DAQ) system include an Arduino Mega microcontroller for robot motion control, a load cell amplifier, an NI USB-6218 DAQ for force measurement, a power supply, and a Dell computer for data logging through Labview. In addition, in the hydrosnake experiment, three 1500 lumen underwater lights are placed in the 1.5 m back from the tail of the model. More details of the model–load cell connection are given as Supplementary Data.

The drag coefficient $C_{D}$, the thrust coefficient $C_{T}$, and the lift coefficient $C_{L}$ are evaluated and reported as follows:

(3) $$\begin{array}{cc} C_{D} & =-C_{T}=\frac{F_{x}}{\frac{1}{2}\rho U^{2}S},\\ C_{L} & =\frac{F_{y}}{\frac{1}{2}\rho U^{2}S}, \end{array}$$

where $F_{x}$ and $F_{y}$ are the measured force, ρ is the fluid density, and we use the wetted surface area $S=0.6138m^{2}$ for the hydrosnake and the wing area $S=s_{f}\times c_{f}=3375m^{2}$ in the denominator for the morphing foil, in which $s_{f}=0.675m$ and $c_{f}=0.5m$ are the length of the span and chord.

### Simulation tool

Fluid simulation has been conducted on morphing foil based on the following averaged continuity and momentum equations for the incompressible flow:

(4) $$\frac{\partial {ū}_{i}}{\partial x_{i}}=0,$$

(5) $$\frac{\partial \left({ū}_{i}\right)}{\partial t}+\frac{\partial }{\partial x_{j}}\left({ū}_{i}{ū}_{j}+\bar{{u\prime }_{i}{u\prime }_{j}}\right)=-\frac{1}{\rho }\frac{\partial \bar{p}}{\partial x_{i}}+\frac{1}{\rho }\frac{\partial {\bar{\tau }}_{ij}}{\partial x_{j}},$$

(6) $${\bar{\tau }}_{ij}=\mu \left(\frac{\partial {ū}_{i}}{\partial x_{j}}+\frac{\partial {ū}_{j}}{\partial x_{i}}\right),$$

where ${\bar{\tau }}_{ij}$ in Eq. (4) are the components of the average viscous force tensor, $\bar{p}$ is the average pressure, $\bar{u}$ are the Cartesian components of the average velocity, $\bar{{u\prime }_{i}{u\prime }_{j}}$ are the Reynolds stresses, and μ is the dynamic viscosity. In addition, we implement the SST k-ω Menter turbulence model^58^ to calculate the boundary layer.

**FIG. 8.**
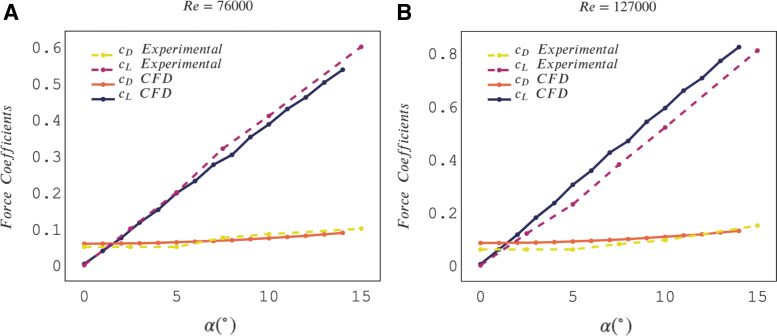
Comparison between 2D simulation and laboratory experiments for a rigid foil at **(A)** Re = 76, 000 and **(B)** Re = 127,000 with various AoAs. AoAs, angles of attack.

For validation, we compare the 2D simulation result against laboratory experiments on a rigid reference foil for Re=76,000 and Re=127,000 and various angles of attack (AoAs). The comparison is shown in Figure 8.

#### Hydrosnake results

**FIG. 9.**
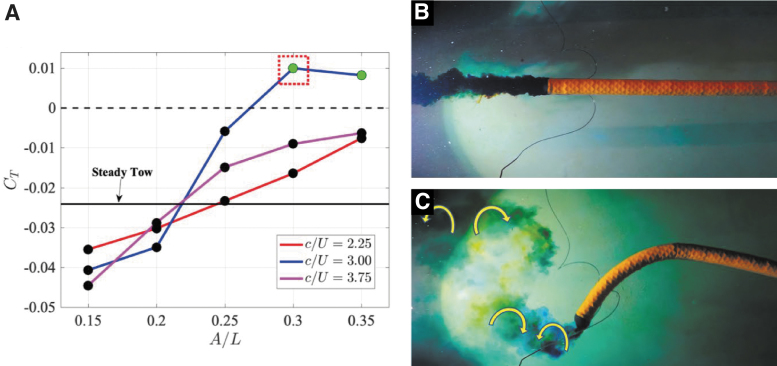
The result of the hydrodynamic experiment of the hydrosnake being towed at U=0.1m∕s. **(A)** The thrust coefficient $C_{T}$ versus commanded $A_{t}∕L$ for various c∕U (λ=L=1.5m). **(B)** The wake pattern of the unactuated robot, represented by the in *black solid line* in the subfigure **(A)**. **(C)** The wake pattern of the actuated hydrosnake with c∕U=3, $A_{t}=0.3L=0.45m$ and λ=L=1.5m, highlighted by the in *dotted red box* in the subfigure **(A)**. Negative $C_{T}$ (*black dots*) are the average drag force on the hydrosnake, whereas positive $C_{T}$ (*green dots*) indicates the average thrust force produced by the robot.

The $C_{T}$ of the hydrosnake being towed at U=0.1m∕s is plotted in Figure 9A. The $C_{T}$ of the unactuated hydrosnake is found to be $C_{T}=-0.0248$ (highlighted as the solid black line in Fig. 9A). At fixed λ=L, $C_{T}$ increases with an increasing tail amplitude ($A_{t}∕L$). Positive $C_{T}$ occurs when the hydrosnake is actuated with c∕U=3.0 (f=0.2Hz) and tail amplitude $A_{t}=0.3L$ and $A_{t}=0.35L$. The hydrosnake can generate thrust in excess of hydrodynamic drag and indicating the robot's potential to reach an average swimming speed of U=0.1m∕s in a free-swimming setting.

We conduct the flow visualization by injecting blue and yellow dye on both sides of the hydrosnake tail. Visualization of the unactuated hydrosnake wake in Figure 9B reveals a narrow wake with mixing due to turbulence. The flow pattern around the tail of the hydrosnake actuated at c∕U=3, $A_{t}=0.3L=0.45m$ and λ=L=1.5m (circled out by the dotted red box in Fig. 9A) is shown in Figure 9C. The coherent vortical structure can be observed with two pairs of vortices, inducing a strong cross-flow jet and shedding into the wake in one period of vibration. A video of the flow visualization corresponding to both Figure 9B and C is provided as Supplementary Data in Supplementary Video S1.

#### Morphing wing results

We test the morphing foil in the towing tank with a similar experimental setup as the hydrosnake. The towing speed is set to be U=0.2m∕s, and hence leads to Re=106,134, given the foil chord length is 507 mm. A rigid foil is constructed and tested as a reference.

**FIG. 10.**
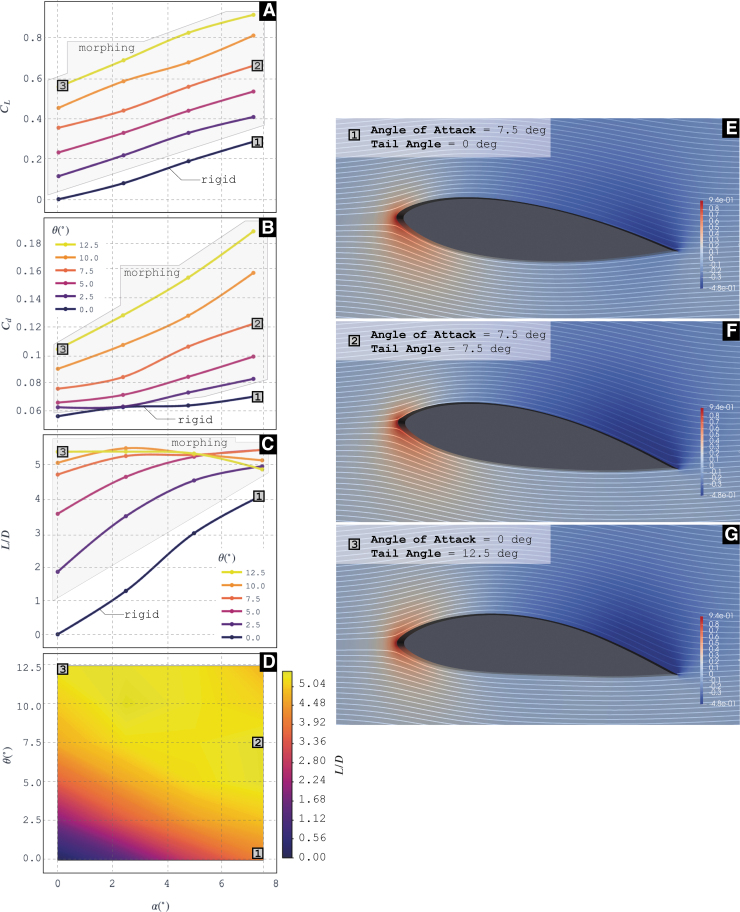
Experimental results and hydrodynamic simulations. We show lift and drag coefficients for the morphing and rigid configuration as well as its L/D ratio for different AoAs. **(A)** Cl—alpha. Experimental. **(B)** Cd—alpha. Experimental. **(C)** L/D—alpha. Experimental. **(D)** Heat map relating tail angle—alpha with values for L/D. **(E)** Pressure field along with streamlines for the rigid airfoil with alpha = 7.5°. **(F**, **G)** Pressure field along with streamlines for morphing configurations with different alpha and tail angles.

Shown in Figure 10, we plot the contour of $C_{D}$ (a), $C_{L}$ (b) and $C_{L}∕C_{D}$ versus α and θ.

## Conclusions and Discussion

In this article, we presented a novel method for rapidly manufacturing flexible robotic platforms for aquatic applications based on discrete lattice materials, consisting of modular mass produced parts that are assembled into voxels and lattices to form 1D, 2D, and 3D structures with programmable mechanical properties. We select a 1D beam and 2D surface to demonstrate that by combining two part types, rigid and flexural, we can design a heterogeneous beam and surface with controlled bending stiffness anisotropy. We then incorporate actuation in the form of a servo with tendons that span the length of the structure, and can create bidirectional continuous shape morphing by pulling either the left or right tendon.

We then assemble actuation units into two discrete cellular soft robotic platforms that can provide smooth continuous shape change through coordinated motion of the distributed actuators: (1) a 1.5 m hydrosnake robot and (2) a 0.5m×0.675m morphing hydrofoil. Finally, we apply skin and the corresponding supporting structures that provide a surface to withstand hydrodynamic pressures while also allowing for shape change without significant wrinkling.

We then measured the hydrodynamic performance of the two systems in a standard towing test. The hydrosnake underwent a wide range of periodic motions designed to mimic anguilliform fish locomotion. The result demonstrates that the robot is able to move naturally in the water and effectively produce net positive thrust with certain prescribed motions. Through a qualitative flow visualization and quantitative hydrodynamic force analysis, the current robotic testing platform, as a simplified version of its abstract biological counterpart, clearly illustrates the nature of the thrust generation of fish undulatory motion and shows a potential to model and construct a freely swimming robot with an improved design based on discrete lattice materials.

Meanwhile, morphing hydrofoil explores heterogeneous lattice surface to construct an out-of-plane morphing of a torsion box, which achieves a camber morphing capability. The result demonstrates that the system is able to achieve same lift-to-drag ratio without drastically changing the AoA of the whole body. Through numerical simulation, the flow visualization reveals a more smooth flow pattern around the morphing foil compared with its rigid counterpart when they achieve the same lift-to-drag ratio.

In summary, this study is the first step of proof-of-concept design and large-scale continuously deforming cellular hydrodynamic robots using a voxel-based construction kit. What this enables are simplified design and construction of user-defined morphing hydrostructures, which can have significant disruptive applications. For example, our technology could be used to reduce the various forms of ship resistance (form, wave, and friction) and hence cut down greenhouse gas emissions. A morphing outer mold line surface could be used to create travelling waves for boundary layer attachment control, which reduces form drag by keeping the laminarized flow attached to the moving surface past the typical separation point.^59^ Wave drag can be reduced through the use of foils, such as those demonstrated at the bow for pitching wave drag reduction.^60^ The ability to morph for retraction, deployment, and AoA control can increase the performance of these structures.

The current hydrodynamic testing demonstrates merely the capability and not the optimality of the hydrodynamic performance of the two systems. Yet, we have seen in nature that aquatic animals can achieve for certain operations a higher performance in producing large thrust forces efficiently.^61^ Therefore, a more systematic investigation^62^ that can use advanced searching algorithms, such as deep reinforcement learning,^63^ is called for to explore the optimal combination of the various input parameters and their effect on the hydrodynamic performance of robotic platforms. In addition, researchers have revealed that the various fins on the fish body play a significant role in manipulating the near flow around body^64^ and hence improving the hydrodynamic efficiency and maneuverability.^65^ Therefore, various shapes of dorsal and caudal fin-inspired devices can be integrated into the current robot design^66^ to improve its hydrodynamic performance.

While the discrete lattice material presented here is mature, both the actuation and skin systems can be improved significantly. The hydrosnake robot was actuated using a servo open-loop system that leads to discrepancies between simulations and the obtained shape underwater. To obtain a greater shape authority on future models, we can explore two solutions. One is using closed-loop systems with higher torque platforms, but with that will come greater mass, which scales almost linearly with required motor torque.^67^ Other interesting alternative is exploring the potential in distributed actuation, which has been tested for discretely assembled microrobots^68^ and larger-scale morphing structures.^69^ The biomechanics of muscle remain constant across length scales,^70^ and while our structural system is scalable, the ability to change shape at larger scales may quickly exceed commercially available centralized actuation sources.

Apart from actuation, the skin is a key component for the design and construction of any aerial/aquatic robot or transportation system to support aerodynamic/hydrodynamic loadings. In this article, a hybrid rib and skin system is used to form a streamlined body and smooth surface for the robot. One of the problems we observed for the hydrosnake is that the currently selected tight skin reflects the underlying feather pattern, which causes drag to increase. In contrast, if a loose skin is chosen wrinkles form during the body deformation, whereas the internally enclosed water that moves together with the body may cause a large skin deformation and even local traveling waves. Investigating new materials, such as hydrogel,^71^ may form new types of artificial skin that mimic nature and improve the robot aerodynamic/hydrodynamic performance.

In addition, microscopic surface patterns inspired by the shark skin surface^72^ can help reduce drag, whereas flexible pressure sensors inspired by the fish lateral line^73^ can be incorporated into the skin and help further enhance the system performance, by allowing the robot to sense the near-body flow and effect closed-loop control of its actuation.^27^

## Supplementary Material

Supplemental data

Supplemental data
