# Supplementary material for: Modular Morphing Lattices for Large-Scale Underwater Continuum Robotic Structures
Source: Soft Robot. 2023 Aug 9;10(4):724–36. doi: 10.1089/soro.2022.0117 (PMC10442689; doi:10.1089/soro.2022.0117)
Supplement: Supplemental data [file Suppl_Data.pdf]

Supplementary Material for:

Modular Morphing Lattices for Large Scale Underwater Continuum Robotic Structures

- 1. Mechanical characterization of the modular lattice
- 2. Hydrosnake design, control and simulation
- 3. Morphing wing design, control and simulation
- 4. Towing carrier details

1. Mechanical characterization of the modular lattice

This tendon actuated robotic structure is actuated using a compliant lattice, capable of large plastic deformation. To better characterize suitable motors for optimal lattice deformation, we built a method to relate strain of the tendon, the tension generated and the centroid deformation of the structure.

Using Grasshopper[1] for geometry generation and Karamba's nonlinear beam solver[2] , we developed a workflow to determine the curvature of the lattice-beam and its inner tendon tension given a certain strain value. To conclude this section, we show a validation platform composed by custom built jigs on an Instron 4411 and a computer vision system made with a Raspberry Pi 4, its camera module and Python-OpenCV.

[Figure 1 about here.]

1.1. Virtual Model

We follow literature for internal tendon-driven manipulators[3,4] to describe the axial load of a tendon given a specific elastic modulus of the beam and desired curvature of the beam. Those cited papers derived the load case of a continuum robot in absence of external loads, resulting in the following relation:

$$\kappa = \frac{1}{R_c} = \frac{d}{EI^*}T$$

We compute the specific elastic modulus (EI^*) of any given n by n voxel beam with a custom Grasshopper - Karamba - Python tool. We simulate a 3-point bending test following the ASTMD4476 standard. Abstracting the voxel beam as a continuum foam beam, using beam theory for simply supported beam length l with a load P at its geometrical center, with a deflection δ, we can obtain the Specific Bending Stiffness EI^* (Nm^2):

$$EI^* = \frac{PL^3}{48\delta}$$

To determine the curvature of an actuated lattice beam, we compute a strain-driven finite element analysis that assumes no tendon elongation. We perform a non-linear analysis using the dynamic relaxation method of Karamba3D.[2] When converged, we run a script to determine the curvature of the virtual centroid of the beam.

With the following method, we can analyze the curvature of a virtual voxel beam at a given actuation strain value.

1.2. Empirical Validation

[Figure 2 about here.]

Here, we validate the virtual model described above. Custom jigs were built to replicate the virtual tests developed and mounted to an Instron 4411.

The jig allows us to install voxel lattices in cantilever and route its tendon to the Instron strain axis. With an Instron tensile testing machine, we determine a strain rate and read the tendon tension values. A wall mounted Raspberry Pi 4 and camera uses computer vision to detect Aruco targets and interpolate the beam's radii and center of deformation, as seen in Fig. 3. The whole system is shown in Fig. 2.

[Figure 3 about here.]

We show here results for a 1 by 3 beam of the virtual model simulation vs. the empirical model test.

[Figure 4 about here.]

2. Hydrosnake design, control and simulation

2.1. Hydrosnake sub-components design

[Figure 5 about here.]

Fig.5-A demonstrates the motion of neighboring ribs resulting in smooth overlap at large strains. This effectively creates a constant tangent surface which the outer skin slides over. This architecture also minimizes the impact of the ribs on the heterogeneous beam core. The lattice rib system is manufactured at its unfolded state by laser cutting 1/32" Delrin sheets.Fig.5-B the unfolded geometry of the lattice rib system for three voxels. The elastic fabric is cut using a Zund large format cutter and sewed in a Brother machine with a zigzag stitch that allows for a longitudinal elongation (Fig.5-F). The details of the internal skin attachments, with velcro on both ends of the body and closing the front and rear strips with lateral zippers, is shown in Fig.5-D.

2.2. Hydrosnake control and simulation

[Figure 6 about here.]

The next challenge was to best fit the ideal splines generated with the equation for backward-traveling wave anguilliform swimmer and out robot. Because of our motion is limited (as Hydrosnake divided in 4 sections with constant curvature) we found the best fit by simulating the snake and making numerical optimization to find the regimes in which we would minimize the discrepancy between the curve and the center line of the robot. The shape to match in this case was a sine with exponential decay.

In Fig.6-A we see snapshots of the snake position over the course of a complete cycle with the overlaid ideal kinematics. While the sign of the snake curvature appears to be in agreement across all snapshots, the amplitude of the positional error grows from the snake head to a maximum at the tail. Fig.6-B explores this further by tracking the time history of the positions of the four sectional endpoints at 25%, 50%, 75% and 100% snake length. The trajectory of the first point nearest the snake head agrees well with the commanded position, but the fourth point at the tail exhibits large discrepancies in amplitude and phase. At the tail both hydrodynamic and inertial forces are greatest and combine to force the observed system response to lag the commanded position by 90 degrees with nearly twice the commanded amplitude. This likely indicates insufficient actuator torque to overcome these external forces.

3. Morphing wing control and simulation

3.1. Morphing wing sub-components design

For the airfoil selection, we designed a variant of an Eppler 838 (renamed in Fig.7 as Eppler21-838 Mod) using XFLR5.[5] In Fig.7 we show all the candidates considered. We increased the thickness close to the trailing edge to delay the boundary layer separation at a high angle of attack but also to make room for an array of 4 voxels inside the wing without becoming too large to test with the available tow tank. The span of the wing is composed of 9 layers of voxels. The leading edge is attached permanently to the first layer of rigid voxels. The skin system is composed of layers of tiled GFRP over a kirigami folded structure (manufactured using a Hylite, an aluminum 3A composite material) with high compressive stiffness, but compliance in bending. This discontinued geometry is needed to have a morphing outer skin, thin enough to overlap with minimal aerodynamic perturbation but stiff enough to keep the overlapping zones motionless under the pressure gradients. The tiles were manufactured with a smaller

radius than the operative value to pre-load them when assembled. Details are shown in Fig. 8

[Figure 7 about here.]

[Figure 8 about here.]

3.2. Morphing wing control and simulation

[Figure 9 about here.]

The first prototype experienced frequent servo failure, which suggested a demand for higher torque motors. That is why we change here from high torque waterproof servos to stepper motors. Dual shaft Nema23 enables the possibility if placed correctly, to pull bidirectionally a heterogeneous beam.

To characterize the number of motors required, we obtain the pressure distribution of the wing using XFLR5 for the worst-case scenario, and an angle of attack of 12.5deg with a tail deflection of 12.5deg.

A detailed structural simulation using Oasys GSA was done implementing the previously obtained pressure distribution with a strain in the opposing direction from the tendon actuation. Fig. 10-B shows that the residual tension of the tendon reached 100N. In conclusion, we could govern the behavior of these three sections with one Nema23 stepper motor, thus, for the whole wing we can use only 3 motors.

The steppers are fixtured inside the first layer of rigid voxels, routing tendons to an aluminum frame that encapsulates the structure. A system to individually pre-stress the tendons was needed to guarantee that backlash won't affect the performance as it is an open loop strain driven model.When installed, the wing was able to perform up to 12.5 angles of rotation continuously without stalling.

3.2.1 Control of kinematics

In order to simulate the morphed state of the wing, related with the deformation of the main core beam, we developed the following relationships. This deformed states will be the one we used to simulate the 2 Dimentional CFD. For the morphing foil system in this paper, the angle of attack α is defined as the angle of rotation of the full-body around the axis of its aerodynamic center. On the contrary, tail angle θ is defined as the one generated by morphing the wing, shown in Fig. 10.

[Figure 10 about here.]

The shape of the morphing foil can be controlled based on its centroid line, assuming constant curvature deflections for tendon actuated foams.[6–9] Shown in Fig. 10, $\forall\theta \in [\frac{\pi}{4}, \frac{-\pi}{4}]$, the coordinate of any given point of the centroid line $p = p(x, y) = p(s, \theta)$ can be defined as follows, for $s \in [a, b)$,

$$\begin{aligned} x &= s \\ y &= 0, \end{aligned} \tag{1}$$

for $s \in [b, c)$,

$$\begin{aligned} x &= \frac{c-b}{\theta} sin(\theta \frac{s-b}{c-b}) + b, \\ y &= \frac{c-b}{\theta} (cos(\theta \frac{s-b}{c-b}) - 1), \end{aligned} \tag{2}$$

and for $s \in [c, d]$,

$$\begin{aligned} x &= sin(\theta - \frac{\pi}{2})(c-s) + \frac{c-b}{\theta} sin(\theta) + b, \\ y &= cos(\theta - \frac{\pi}{2})(cs) + \frac{c-b}{\theta} (cos(\theta) - 1). \end{aligned} \tag{3}$$

4. Towing carrier details

Here we describe the beam system designed to join the head of the robot with the carrier and read the loads that the robot experiences. The structure consists of a core 8020 aluminum beam combined with additional attachments shown in the exploded view in Fig. 11. Starting from the bottom, we find the leading edge cap of the robot. This element is fabricated in two pieces from 3D printed PLA. The lower part constrains the vertical position of the beam with respect to the head while the upper element constrains the horizontal position. This upper element also supports the front strip of skin material.

[Figure 11 about here.]

At the base of the robot head, an aluminum plate serves as a mechanical interface between the

first voxel of the robot and the base of the 8020. Fittings were developed to allow tooling access to the core of the 8020 and the ATI Gamma load cell attachment points. Once the load cell is attached to 8020 beams on both sides, the structure is attached to the tow tank carriage with two angular fittings (Fig. 11-B).

References

[1] McNeel., "Grasshopper - generative modeling for rhinoceros.," vol. http://grasshopper.rhino3d.com/.

[2] C. Preisinger, "Karamba, linking structure and parametric geometry," *Archit Design, 2013*, vol. 83: 110-113., 2013.

[3] D. B. Camarillo, C. F. Milne, C. R. Carlson, M. R. Zinn, and J. K. Salisbury, "Mechanics modeling of tendon-driven continuum manipulators," *IEEE Transactions on Robotics*, vol. 24, no. 6, pp. 1262–1273, 2008.

[4] P. Rao, Q. Peyron, S. Lilge, and J. Burgner-Kahrs, "How to model tendon-driven continuum robots and benchmark modelling performance," *Frontiers in Robotics and AI*, vol. 7, 02 2021.

[5] M. D. A. Deperrois and H. Youngren, "Xflr5 is an analysis tool for airfoils, wings and planes operating at low reynolds numbers.," vol. http://www.xflr5.tech/xflr5.htm.

[6] I. Gravagne, C. Rahn, and I. Walker, "Large deflection dynamics and control for planar continuum robots," *IEEE/ASME Transactions on Mechatronics*, vol. 8, no. 2, pp. 299–307, 2003.

[7] I. Gravagne and I. Walker, "On the kinematics of remotely-actuated continuum robots," in *Proceedings 2000 ICRA. Millennium Conference. IEEE International Conference on Robotics and Automation. Symposia Proceedings (Cat. No.00CH37065)*, vol. 3, pp. 2544–2550 vol.3, 2000.

[8] B. Jones and I. Walker, "Kinematics for multisection continuum robots," *IEEE Transactions on Robotics*, vol. 22, no. 1, pp. 43–55, 2006.

[9] M. Rolf and J. J. Steil, "Constant curvature continuum kinematics as fast approximate model for the bionic handling assistant," in *2012 IEEE/RSJ International Conference on Intelligent Robots and Systems*, pp. 3440–3446, IEEE, 2012.

List of Figures

1 A) Parametric geometry generation. B) FEM analysis of the lattice-beam. 6

2 A)Platform to test beams to find strain - tension - curvature correlations. The tendon (magenta) is routed orthogonal to met the Instron requirement. B)Raspberry Pi 4 mounted in the wall with the camera module used to track motion. C)Instrument layout. 1- Cantilever tooling , 2-Instron 4411 with tooling mounted, 3- Vision System, 4- Control station. 7

3 OpenCV-Python artificial vision analyzer to determine radius of centroid given a determined tendon strain. 8

4 Simulations vs Testing results. 9

5 Lattice rib system. A) Neighboring rib panels accommodate shape change and avoid interference by sliding past each other. B) An unrolled lattice rib panel for laser cutting. Skin construction. C) Robot skin. Stitched at the left and flat pattern on the right. D) Detail of the tail enclosure. E) Three different candidates proposed for the skin. F)Zig zag stitch used to allow the elastic fabric to elongate G) Skin over the robot. Note that the perimeter of the skin is less than the cross section perimeter of the robot to force the fabric to work preloaded. H) Robot with the skin. 10

6 Robot shape control evaluation. A) Time steps from dynamic motion in still water, with corresponding simulated shape overlaid. B) Section endpoint (1-4) y position vs. time, comparing experiment and simulation. 11

7 Eppler and NACA candidates. Mod surnames indicates they have been custom modified by us to delay boundary layer separation at higher Angles of Attack. 12

8 A) Voxel torsion box. B) Adding inverted hexagon. C) Adding glass fiber skin. D) Folded trailing edge. E) Complete assembly before testing. 13

9 A) Airfoil morphed shapes for different centroid curvatures. B) Worst case FEM analysis using Oasys GSA. Colored, axial stresses for 1/3 of the wing section 14

10 A) Airfoil morphed shapes for different centroid curvatures. B) Worst case FEM analysis using Oasys GSA. Colored, axial stresses for 1/3 of the wing section 15

11 Robot - Carriage Assembly System A) Prototype ready to go to the water. B) Exploded view of the current design. (1) 8020 1530 aluminum beam. (2) First voxel of the robot. (3) 1/4" waterjet aluminum plate. (4) PLA 3d printed leading edge. (5) Lower fitting. 1/4” waterjet aluminum plate. (6) ATI Gamma Sensor IP68. (7) Upper fitting. 1/4” waterjet aluminum plate. Towing Carrier Details. C). Wing in the water at =10 deg. D) 1. Control station (not visible, beneath the gantry). Power supply, micro-controller, computer, stepper drivers and cooling station. 2. Towing carriage. 3. 6 axis load cell. 4. Wing. E) Full range of the carrier. F) Detailed join between the 8020 beam and the carrier attachment point. 16

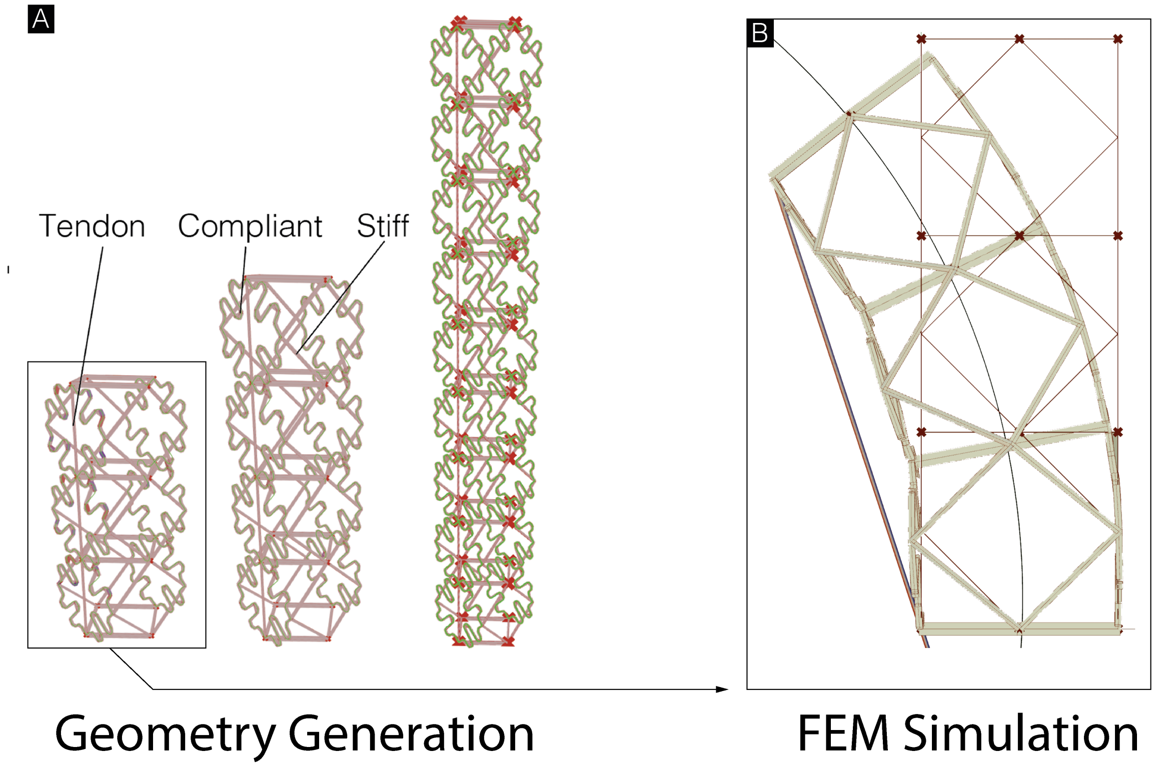

Figure 1. A) Parametric geometry generation. B) FEM analysis of the lattice-beam.

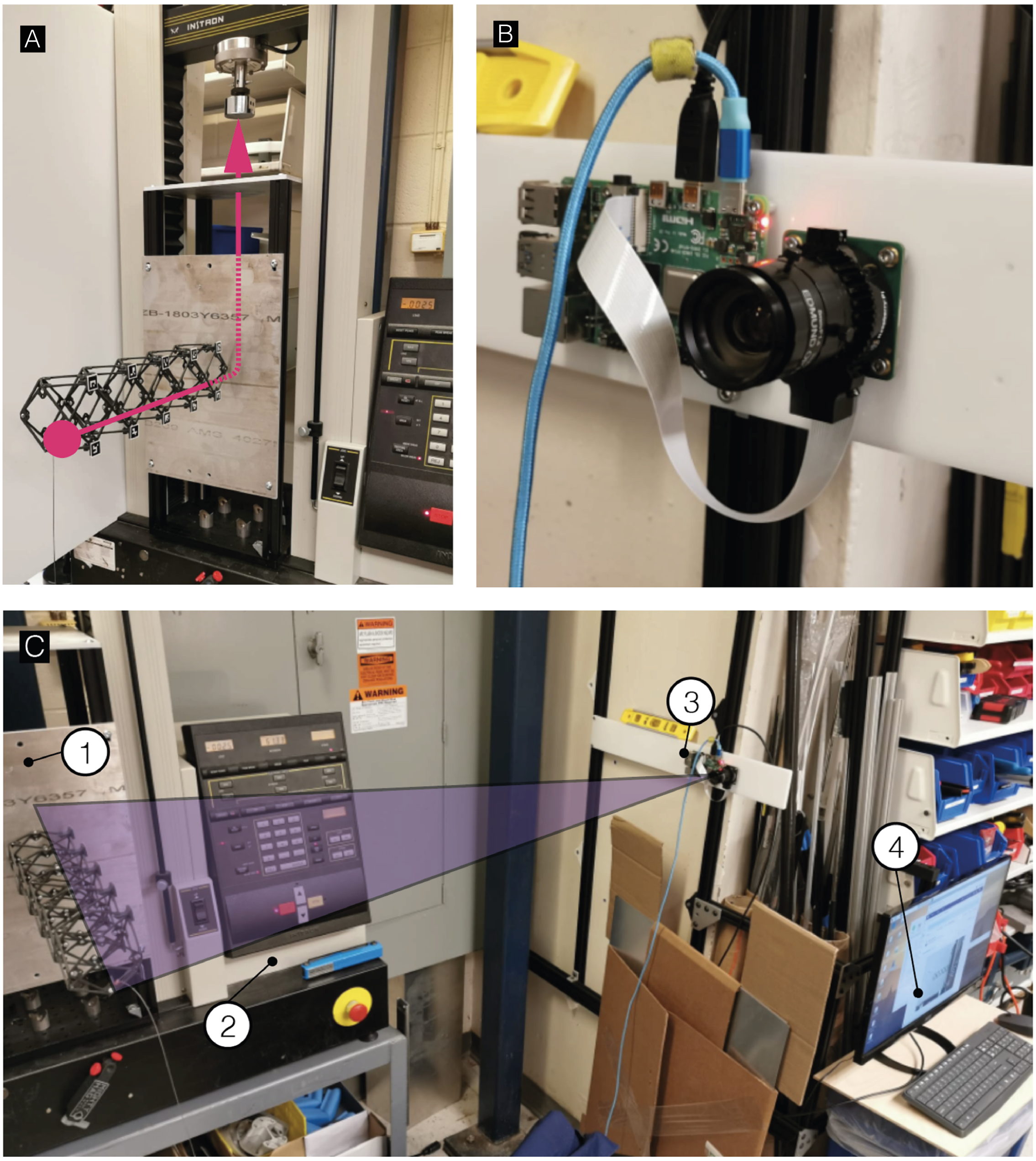

Figure 2. A)Platform to test beams to find strain - tension - curvature correlations. The tendon (magenta) is routed orthogonal to met the Instron requirement. B)Raspberry Pi 4 mounted in the wall with the camera module used to track motion. C)Instrument layout. 1- Cantilever tooling , 2-Instron 4411 with tooling mounted, 3- Vision System, 4- Control station.

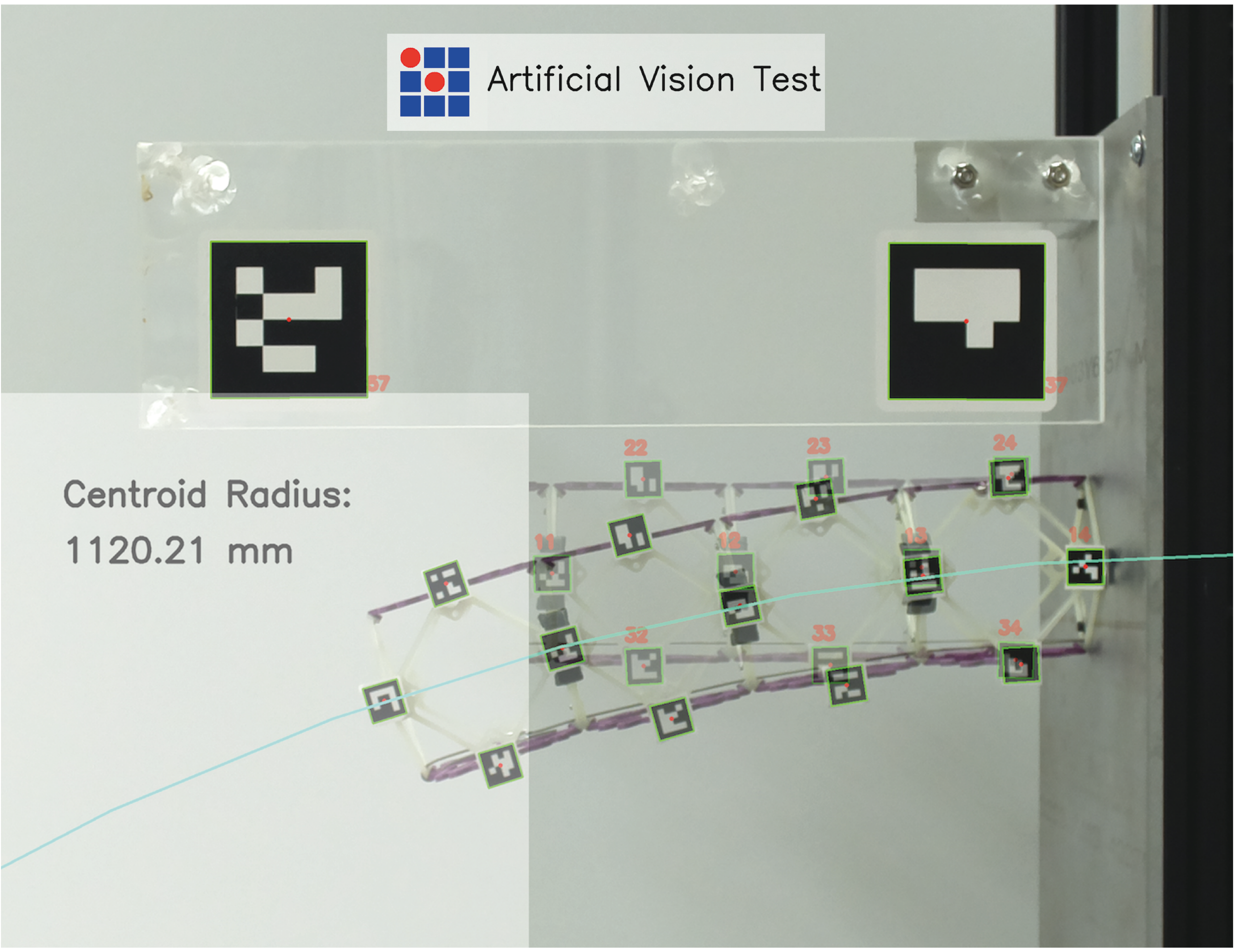

Figure 3. OpenCV-Python artificial vision analyzer to determine radius of centroid given a determined tendon strain.

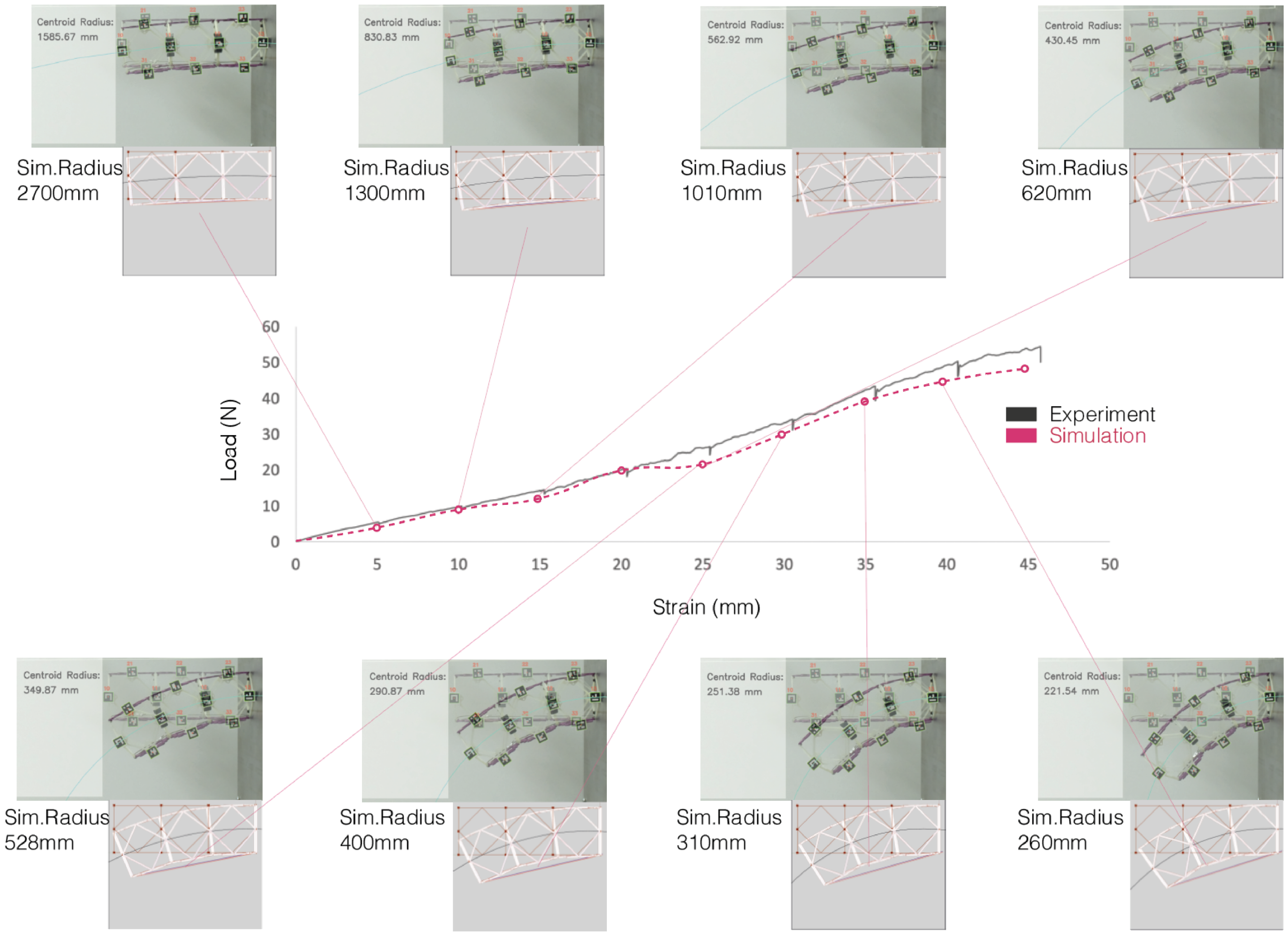

Figure 4. Simulations vs Testing results.

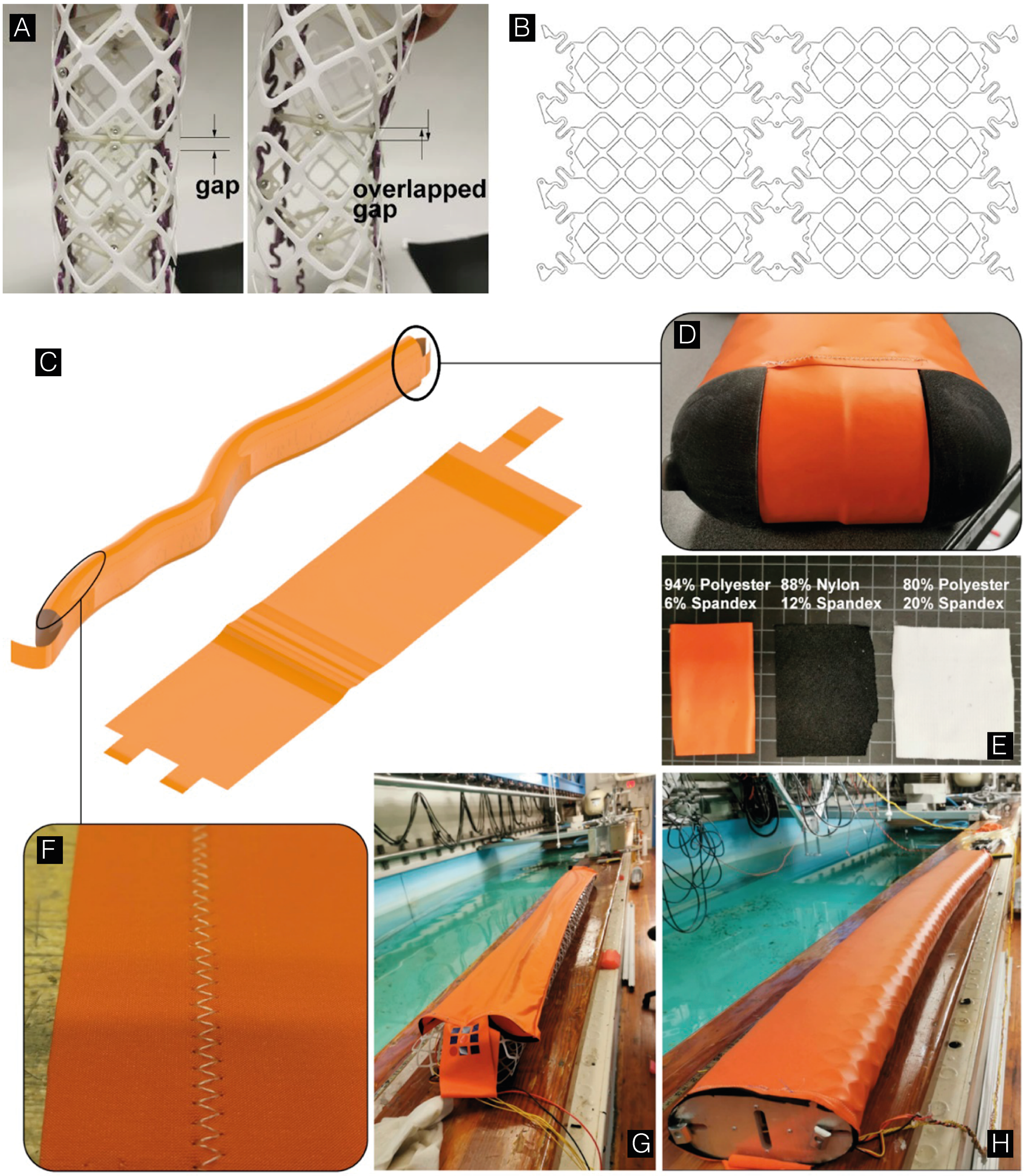

Figure 5. Lattice rib system. A) Neighboring rib panels accommodate shape change and avoid interference by sliding past each other. B) An unrolled lattice rib panel for laser cutting. Skin construction. C) Robot skin. Stitched at the left and flat pattern on the right. D) Detail of the tail enclosure. E) Three different candidates proposed for the skin. F)Zig zag stitch used to allow the elastic fabric to elongate G) Skin over the robot. Note that the perimeter of the skin is less than the cross section perimeter of the robot to force the fabric to work preloaded. H) Robot with the skin.

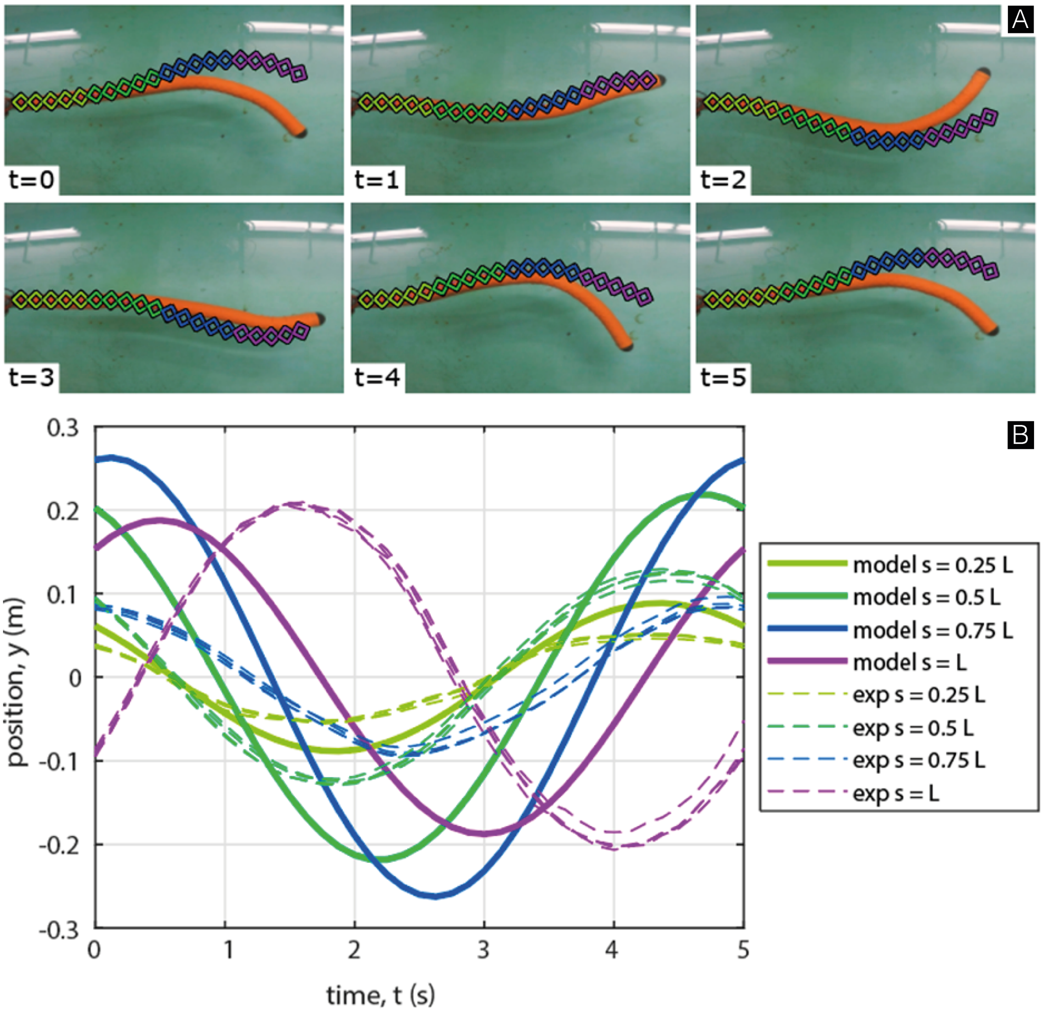

Figure 6. Robot shape control evaluation. A) Time steps from dynamic motion in still water, with corresponding simulated shape overlaid. B) Section endpoint (1-4) y position vs. time, comparing experiment and simulation.

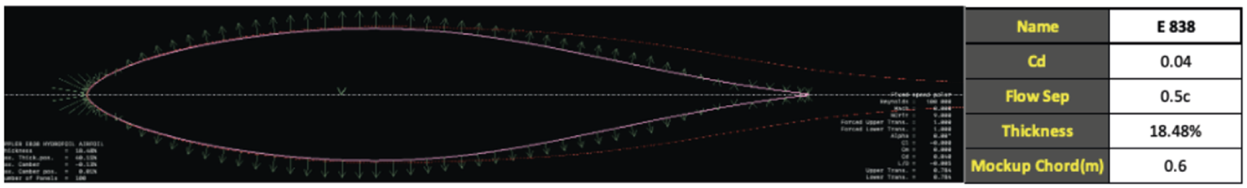

Name	E 838
Cd	0.04
Flow Sep	0.5c
Thickness	18.48%
Mockup Chord(m)	0.6

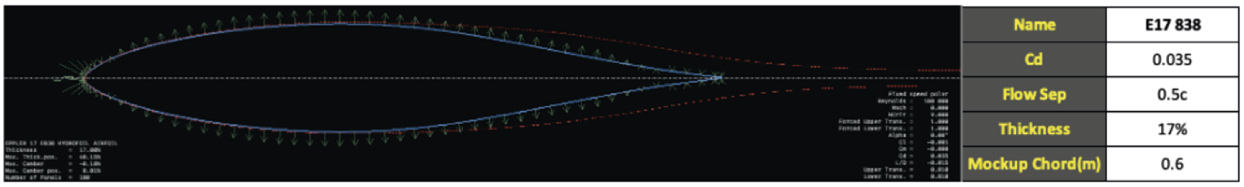

Name	E17 838
Cd	0.035
Flow Sep	0.5c
Thickness	17%
Mockup Chord(m)	0.6

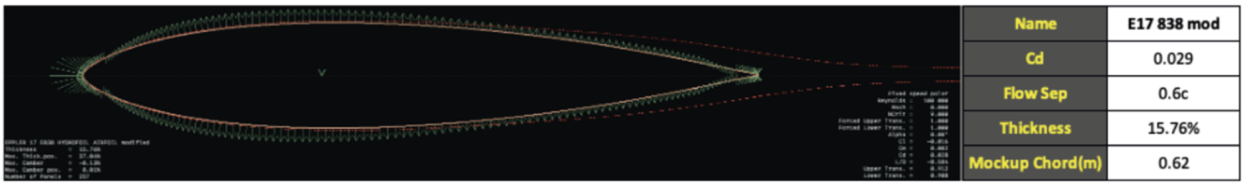

Name	E17 838 mod
Cd	0.029
Flow Sep	0.6c
Thickness	15.76%
Mockup Chord(m)	0.62

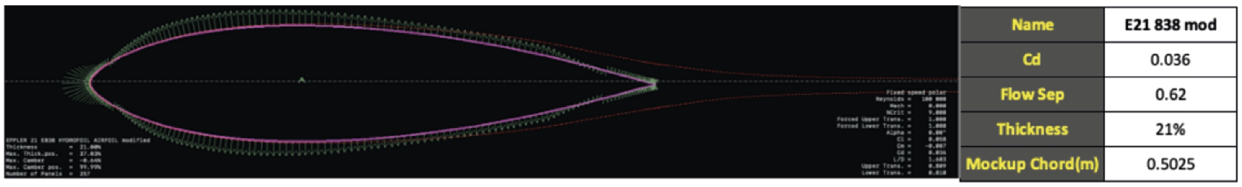

Name	E21 838 mod
Cd	0.036
Flow Sep	0.62
Thickness	21%
Mockup Chord(m)	0.5025

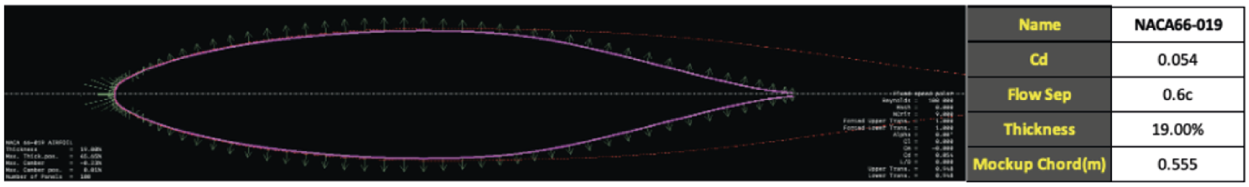

Name	NACA66-019
Cd	0.054
Flow Sep	0.6c
Thickness	19.00%
Mockup Chord(m)	0.555

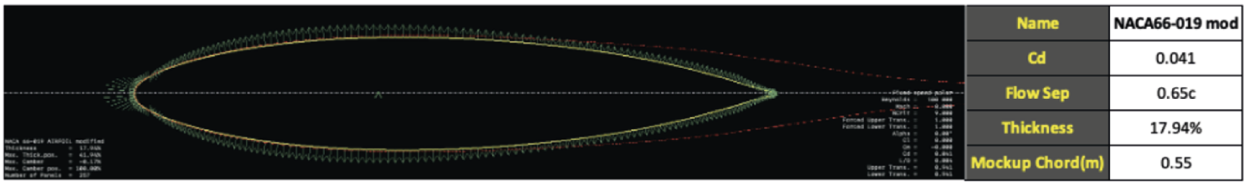

Name	NACA66-019 mod
Cd	0.041
Flow Sep	0.65c
Thickness	17.94%
Mockup Chord(m)	0.55

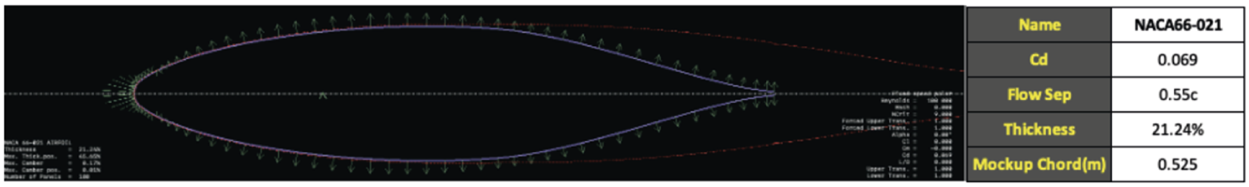

Name	NACA66-021
Cd	0.069
Flow Sep	0.55c
Thickness	21.24%
Mockup Chord(m)	0.525

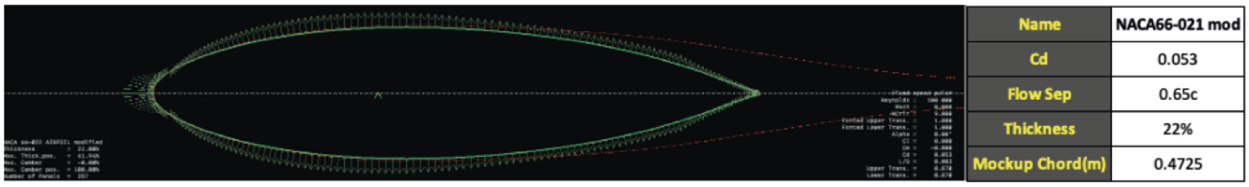

Name	NACA66-021 mod
Cd	0.053
Flow Sep	0.65c
Thickness	22%
Mockup Chord(m)	0.4725

Figure 7. Eppler and NACA candidates. Mod surnames indicates they have been custom modified by us to delay boundary layer separation at higher Angles of Attack.

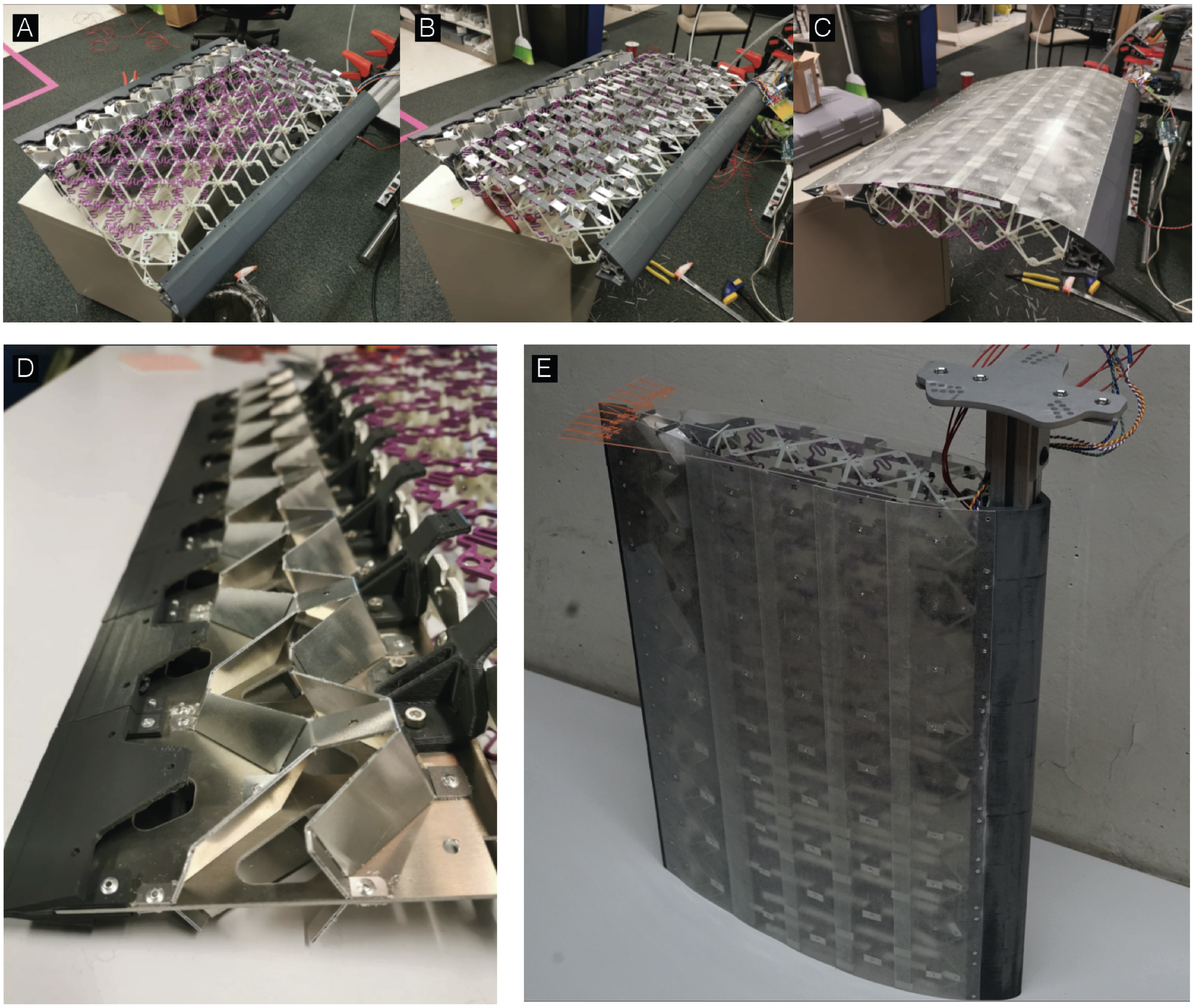

Figure 8. A) Voxel torsion box. B) Adding inverted hexagon. C) Adding glass fiber skin. D) Folded trailing edge. E) Complete assembly before testing.

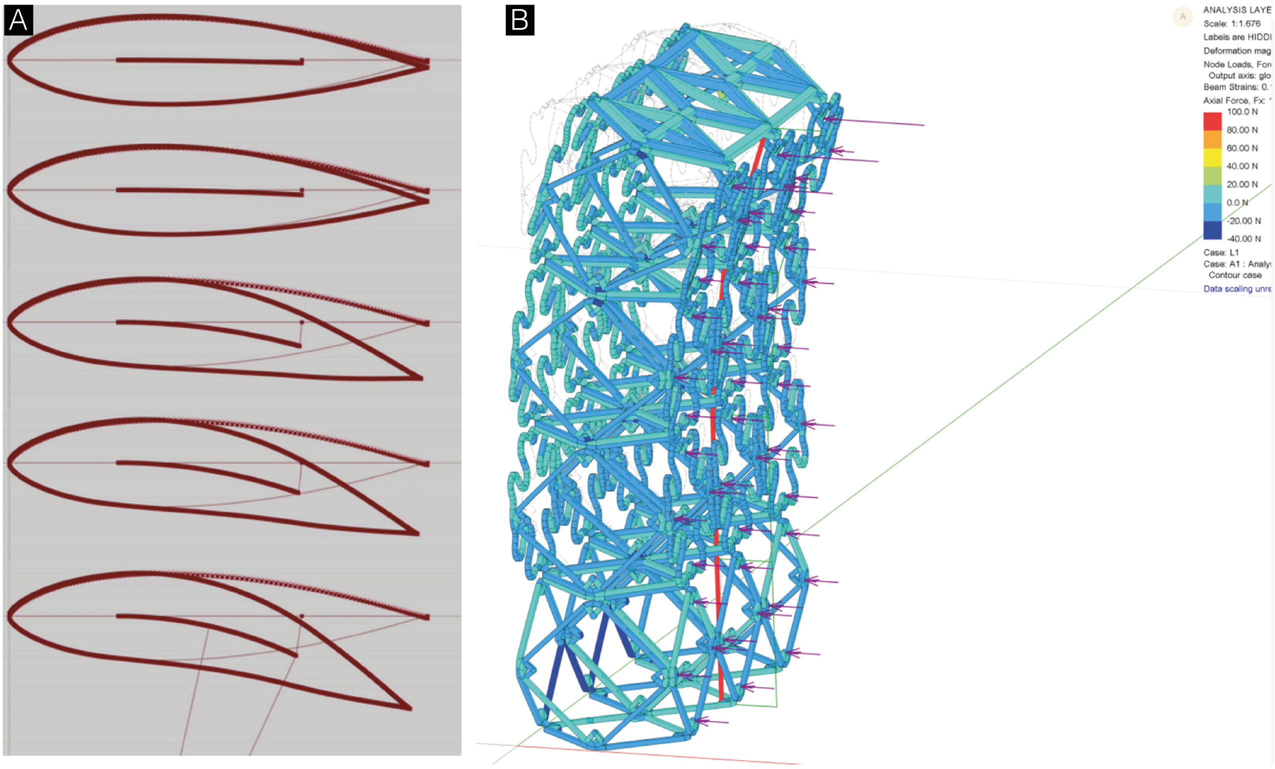

Figure 9. A) Airfoil morphed shapes for different centroid curvatures. B) Worst case FEM analysis using Oasys GSA. Colored, axial stresses for 1/3 of the wing section

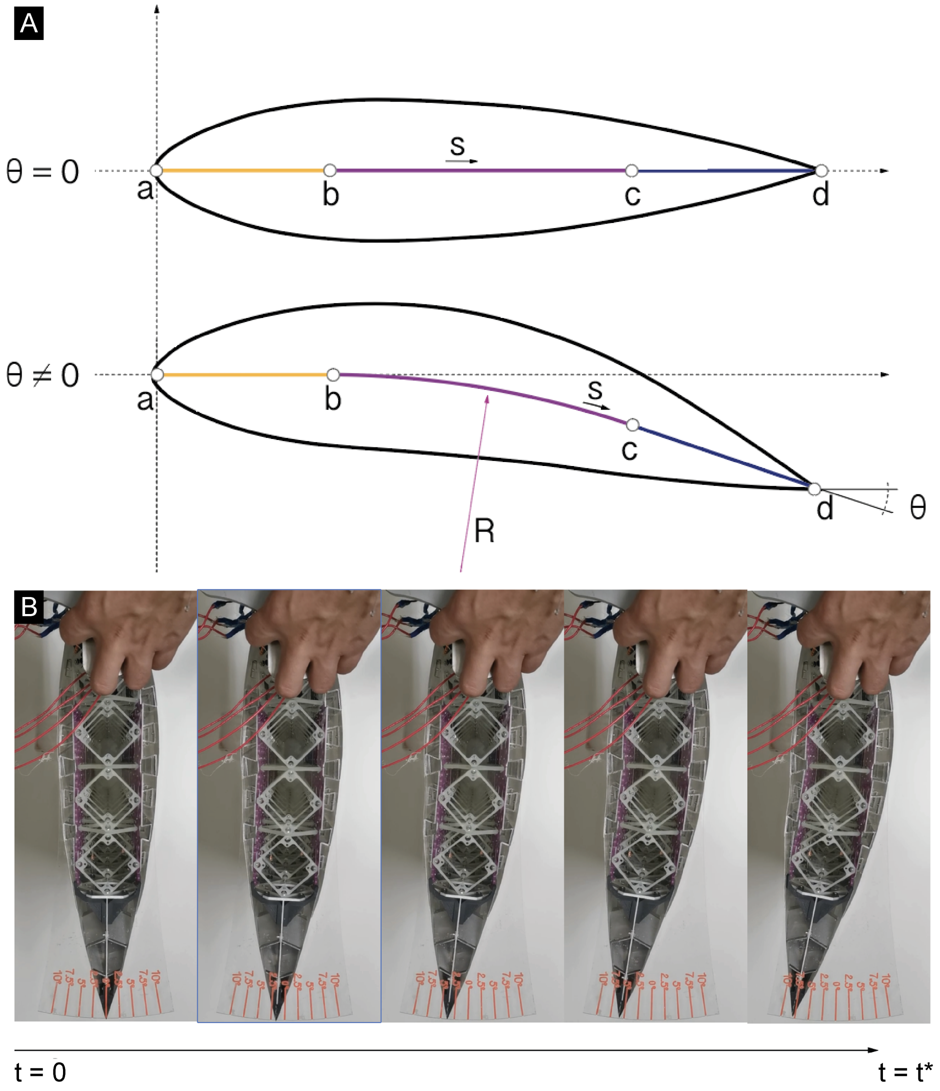

Figure 10. A) Airfoil morphed shapes for different centroid curvatures. B) Worst case FEM analysis using Oasys GSA. Colored, axial stresses for 1/3 of the wing section

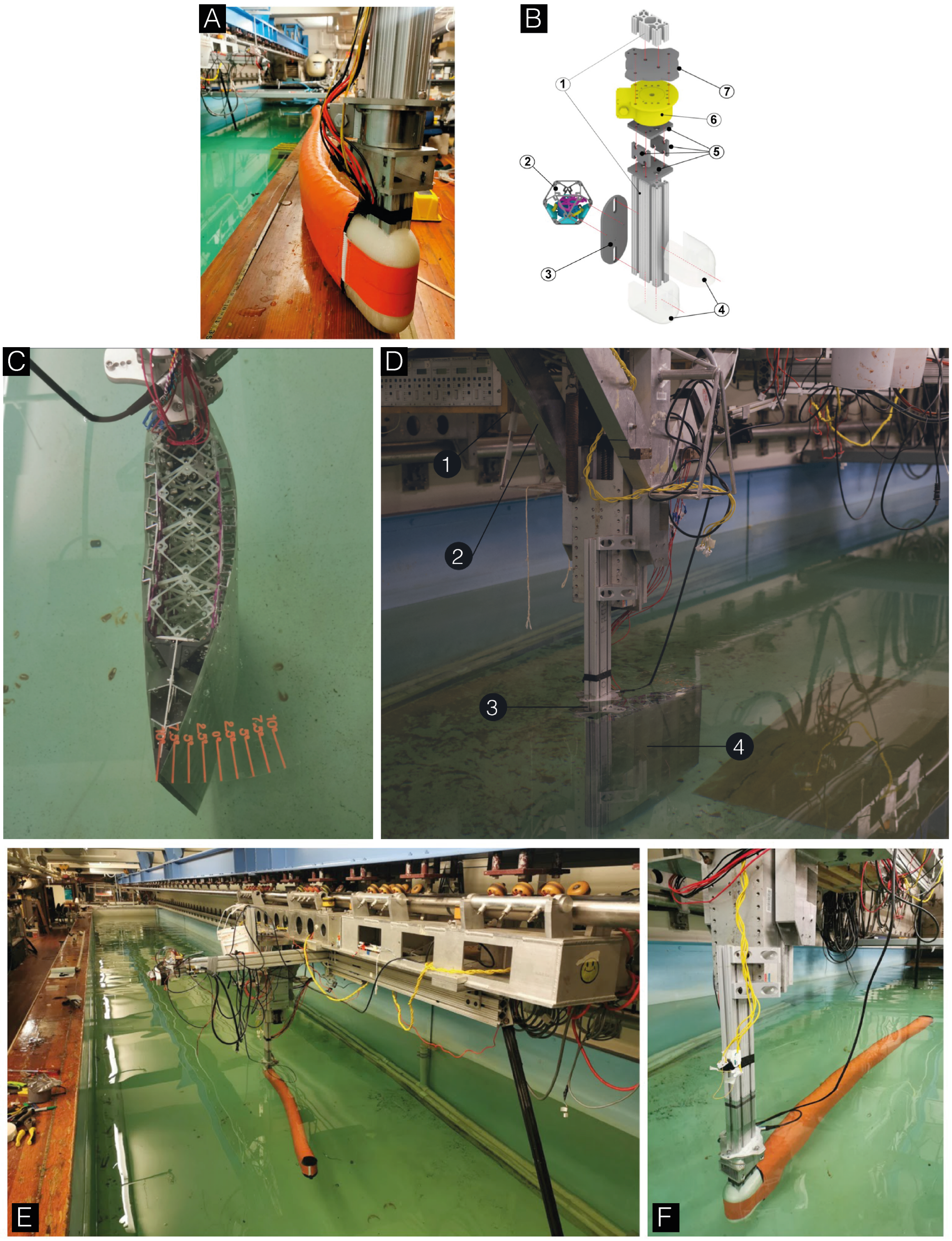

Figure 11. Robot - Carriage Assembly System A) Prototype ready to go to the water. B) Exploded view of the current design. (1) 8020 1530 aluminum beam. (2) First voxel of the robot. (3) 1/4" waterjet aluminum plate. (4) PLA 3d printed leading edge. (5) Lower fitting. 1/4" waterjet aluminum plate. (6) ATI Gamma Sensor IP68. (7) Upper fitting. 1/4" waterjet aluminum plate. Towing Carrier Details. C). Wing in the water at =10 deg. D) 1. Control station (not visible, beneath the gantry). Power supply, micro-controller, computer, stepper drivers and cooling station. 2. Towing carriage. 3. 6 axis load cell. 4. Wing. E) Full range of the carrier. F) Detailed join between the 8020 beam and the carrier attachment point.
